# Identification of novel prognostic targets in glioblastoma using bioinformatics analysis

**DOI:** 10.1186/s12938-022-00995-8

**Published:** 2022-04-18

**Authors:** Xiaofeng Yin, Quansheng Wu, Zheng Hao, Laizhao Chen

**Affiliations:** Department of Neurosurgery, Second Hospital of Shanxi Medical University, No.382 Wuyi Road, Taiyuan, 030000 Shanxi China

**Keywords:** Glioblastoma multiform, Bioinformatics analysis, Prognosis, Biomarker

## Abstract

**Background:**

Glioblastoma (GBM) is the most malignant grade of glioma. Highly aggressive characteristics of GBM and poor prognosis cause GBM-related deaths. The potential prognostic biomarkers remain to be demonstrated. This research builds up predictive gene targets of expression alterations in GBM utilizing bioinformatics analysis.

**Methods and results:**

The microarray datasets (GSE15824 and GSE16011) associated with GBM were obtained from Gene Expression Omnibus (GEO) database to identify the differentially expressed genes (DEGs) between GBM and non-tumor tissues. In total, 719 DEGs were obtained and subjected to Gene Ontology (GO) and Kyoto Encyclopedia of Genes and Genomes (KEGG) for function enrichment analysis. Furthermore, we constructed protein–protein Interaction (PPI) network among DEGs utilizing Search Tool for the Retrieval of Interacting Genes (STRING) online tool and Cytoscape software. The DEGs of degree > 10 was selected as hub genes, including 73 upregulated genes and 21 downregulated genes. Moreover, MCODE application in Cytoscape software was employed to identify three key modules involved in GBM development and prognosis. Additionally, we used the Gene expression profiling and interactive analyses (GEPIA) online tool to further confirm four genes involving in poor prognosis of GBM patients, including interferon-gamma-inducible protein 30 (IFI30), major histocompatibility complex class II-DM alpha (HLA-DMA), Prolyl 4-hydroxylase beta polypeptide (P4HB) and reticulocalbin-1 (RCN1). Furthermore, the correlation analysis indicated that the expression of IFI30, an acknowledged biomarker in glioma, was positively correlated with HLA-DMA, P4HB and RCN1. RCN1 expression was positively correlated with P4HB and HLA-DMA. Moreover, qRT-PCR and immunohistochemistry analysis further validated the upregulation of four prognostic markers in GBM tissues.

**Conclusions:**

Analysis of multiple datasets combined with global network information and experimental verification presents a successful approach to uncover the risk hub genes and prognostic markers of GBM. Our study identified four risk- and prognostic-related gene signatures, including IFI30, HLA-DMA, P4HB and RCN1. This gene sets contribute a new perspective to improve the diagnostic, prognostic, and therapeutic outcomes of GBM.

## Introduction

Glioblastoma multiform (GBM), the most common brain cancer in central nervous system (CNS), is identified as grade IV glioma, with approximately 5.36 new cases per 100,000 population [1–3]. Due to its highly aggressive characteristics, a 5-year overall survival (OS) of GBM patients treated with maximum surgical excision plus chemoradiotherapy ranges from 0.01 to 29.1% [4, 5]. Surgical resection followed by adjuvant temozolomide-based chemotherapy and radiotherapy is the current feasible treatment of GBM patients [6]. A subset of GBM displays a high heterogeneity, which is strongly associated with morphology characteristics, molecular changes and immunotherapy [1]. Defining the molecular targets for diagnosis and reexamination is crucial for therapeutic action and prognostic outcome of GBM patients.

Several studies has described that significant molecular biomarkers in GBM were identified as prognostic or therapeutic factors, such as O6-methylguanine-DNA methyltransferase (MGMT), epidermal growth factor receptor (EGFR) and isocitrate dehydrogenase (IDH) [7, 8]. GBM patients with IDH wild-type anaplastic astrocytoma had poor overall survival that that in patients with IDH mutant type [9]. According to the classification of CNS tumors, IDH status should be included in the diagnosis of glioma. In addition, IDH mutation inhibitors are effective against IDH1/2 mutated gliomas [10]. Although phase I clinical trials demonstrated a promising antitumor activity, further evidence indicated the limitations of IDH1-mutant inhibitors against glioma growth. A study demonstrated that an inhibitor targeting IDH1-mutant conferred radiation resistance on IDH1-mutant tumor cells [11]. The identification of novel biomarkers may be helpful to improve the clinical outcome of GBM patients and provide a combined approach.

Bioinformatics analysis is a well-orchestrated tool for screening tumor-specific genes and prognosis-relevant biomarkers, which can contribute to the development of cancer treatment [12]. At present, microarray and RNA-seq data downloaded from Gene Expression Omnibus (GEO) database can be used to detect genes transcription expression levels and to provide the technical support for monitoring mRNA expression and cell function prediction [13]. For example, Alshabi et al. reported that high levels of RPL36A and AP1S1 were associated with poor prognosis and pathogenesis of GBM by analyzing lin7A-silenced data samples [14]. Zhou et al. have reported that the expression of RRM2 and CEP55 is responsible for prognosis of GBM [15].

Although numerous reports have identified differentially expressed genes in GBM tumorigenesis via bioinformatic analysis, the prognostic value of these genes has not become widely acceptable in clinical practice. Systematic study of identifying multiple prognostic genes will provide better understanding of GBM therapeutic targets, prognostic judgment and illness monitoring. The aim of present study was to characterize the differentially expressed genes in GBM datasets and identify more genes with prognostic value.

Here, we analyzed the GBM profiles that were downloaded from the GEO database and performed the identification of differentially expressed genes (DEGs) between the GBM tissues and the non-tumor brain tissues. Afterwards, Gene ontology (GO) terms and Kyoto Encyclopedia of Gene and Genome (KEGG) pathways associated with DEGs were explored to elucidate the gene enrichment in GBM. Moreover, protein–protein interaction (PPI) networks and the hub genes were identified according to degree parameters and clustering coefficient. Furthermore, six hub genes were subject to prognostic analysis. Through an integrative bioinformatics strategy, we identified four novel biomarkers (IFI30, HLA-DMA, P4HB and RCN1) as the interests of GBM prognosis assessment. Moreover, the expression of candidates genes in GBM tissues were assessed via qRT-PCR and immunohistochemistry analysis. In short, these results indicated that the four genes might have potential value in prognosis and diagnosis of GBM.

## Results

### Expression analysis of DEGs in GBM

**Fig. 1:**
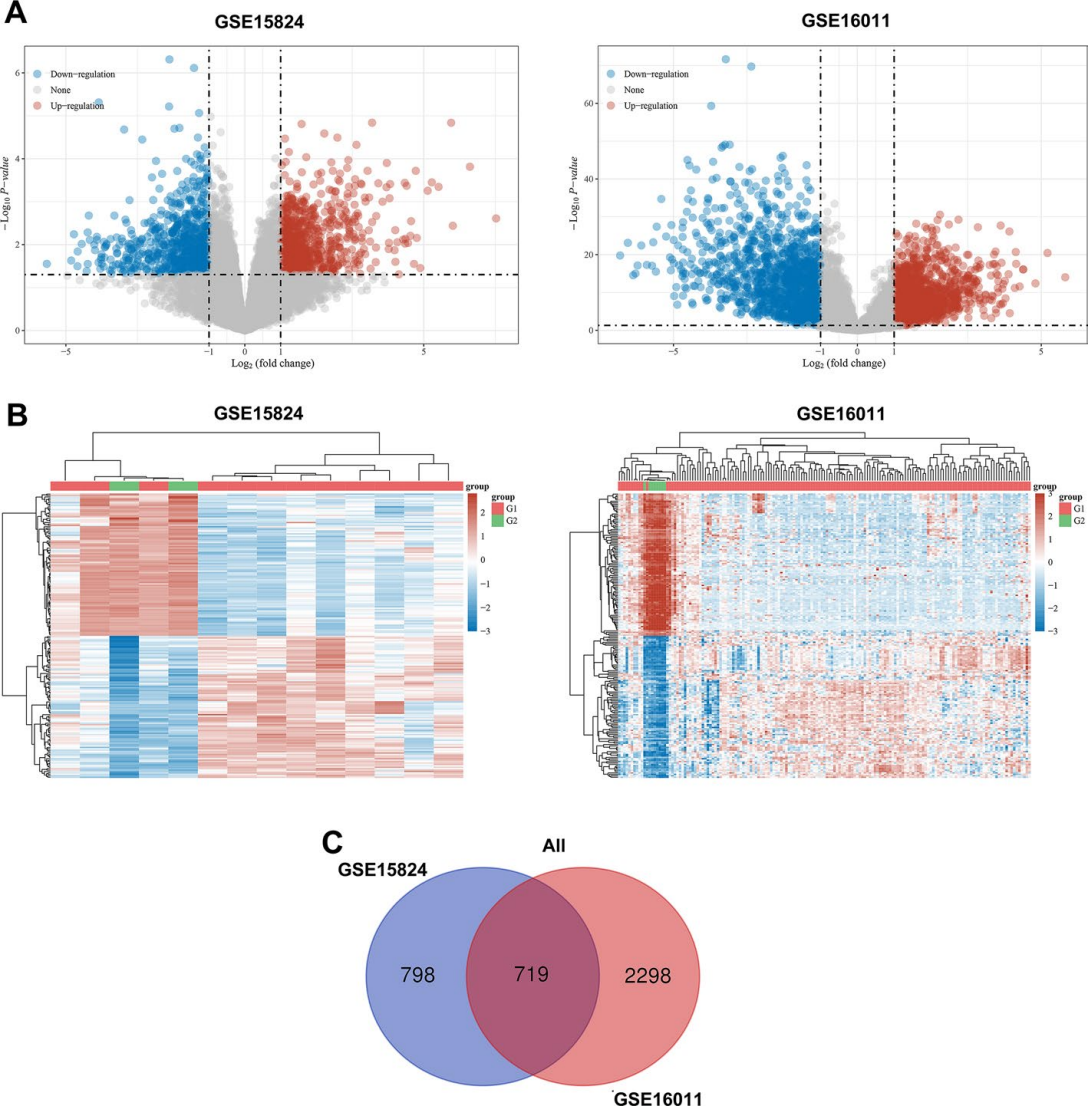
Identification of differentially expressed genes (DEGs) in GBM. **A** Volcano plots of GSE15824 and GSE16011 were analyzed using log_2_ FC > 2 and adjusted *p*-value < 0.05. Upregulated DEGs were shown in red and downregulated DEGs were shown in blue. **B** Hierarchical clustering analysis showing DEGs between GBM and normal tissues. **C** Intersection of all DEGs (*n* = 719) among the expression profiling of GSE15824 and GSE16011

GBM gene expression profiles from the GEO database, GSE15824 and GSE16011, including 10 non-tumor brain samples and 167 GBM tissue samples, were subjected to identification of DEGs. We identified DEGs between GBM samples and non-tumor samples with the cut-off of log2 (Fold Change) (log2|FC|) > 2 and adjusted *p* < 0.05. In GSE15824 dataset, 1517 DEGs were screened out, including 723 downregulated and 794 upregulated genes in GBM samples. Of 3017 DEGs in GSE16011 dataset, 1471 were downregulated and 1546 were upregulated. The volcano plots presented the distributions of differentially expressed genes (Fig. 1A). The heat maps demonstrated the gene expression levels in two datasets (Fig. 1B). Subsequently, the intersection of DEGs in these two datasets was analyzed using Draw Venn Diagram online tools (http://bioinformatics.psb.ugent.be/webtools/Venn/). As shown in Fig. 1C, a total of 719 DEGs were identified in GBM tissues among the two datasets, as compared to the non-tumor tissues.

### Functional enrichment analysis of DEGs in GBM

**Fig. 2:**
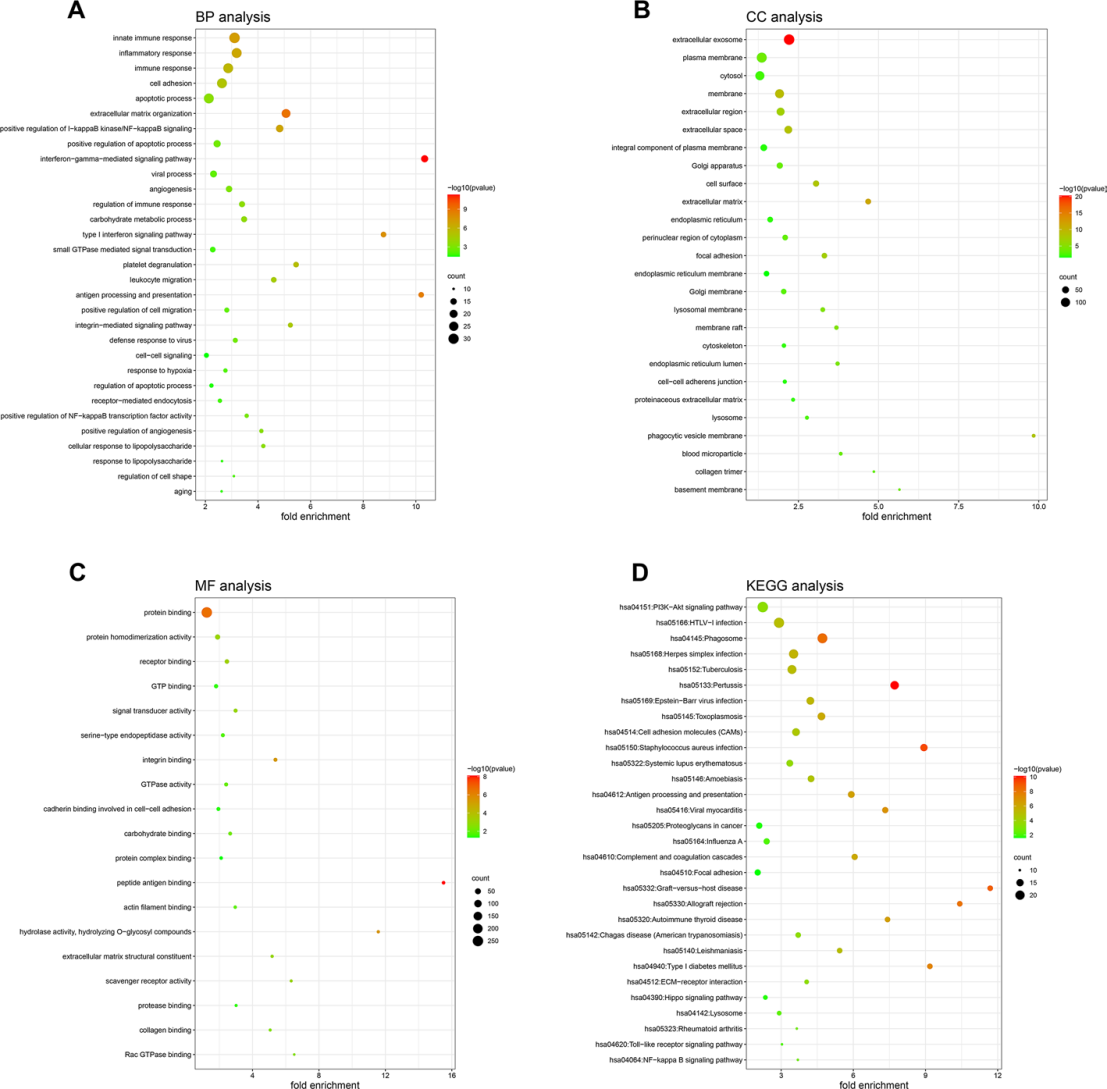
Functional enrichment analysis of DEGs in GBM. **A**–**C** Gene ontology (GO) analysis of DEGs. **D** Kyoto encyclopedia of genes and genomes (KEGG) pathway analysis of DEGs

Function annotation analysis of the 719 DEGs was performed utilizing DAVID online tool [16]. The upregulated DEGs were significantly enriched in the biological processes (BP): innate immune response, inflammatory response, cell adhesion, apoptotic process and positive regulation of NF-kB signaling (Fig. 2A). The upregulated DEGs were significantly enriched in the cellular component (CC): extracellular exosome, plasma membrane, cytosol and extracellular matrix (Fig. 2B). The upregulated DEGs were significantly enriched in the molecular function (MF): protein binding, protein homodimerization activity, receptor binding and signal transducer activity (Fig. 2C). Moreover, KEGG pathway enrichment analysis indicated that the upregulated DEGs were involved in PI3K–AKT signaling pathway, cell adhesion molecules, Hippo signaling pathway, Toll-like receptor signaling pathway and NF-kB signaling pathway (Fig. 2D). In addition, the GO enrichment of downregulated DEGs is shown in Table 1.

**Table 1:** GO and pathway enrichment analysis of downregulated DEGs in GBM

Category	Term	Count, *n*	*p* value
GOTERM_BP_DIRECT	GO:0,043,547 ~ positive regulation of GTPase activity	17	0.0099
	GO:0,035,556 ~ intracellular signal transduction	15	0.0027
	GO:0,007,155 ~ cell adhesion	14	0.0192
	GO:0,006,468 ~ protein phosphorylation	13	0.0387
GOTERM_CC_DIRECT	GO:0,016,021 ~ integral component of membrane	99	0.0019
	GO:0,005,886 ~ plasma membrane	90	4.13E−05
	GO:0,005,622 ~ intracellular	31	0.013
	GO:0,005,887 ~ integral component of plasma membrane	30	0.043
GOTERM_MF_DIRECT	GO:0,005,524 ~ ATP binding	32	0.0383
	GO:0,005,509 ~ calcium ion binding	18	0.0409
	GO:0,004,674 ~ protein serine/threonine kinase activity	12	0.0261
	GO:0,004,672 ~ protein kinase activity	11	0.0437
KEGG_PATHWAY	hsa04144:Endocytosis	9	0.0211
	hsa04010:MAPK signaling pathway	9	0.0273
	hsa04723:Retrograde endocannabinoid signaling	8	0.00055
	hsa04921:Oxytocin signaling pathway	7	0.0198

### PPI network and hub gene analysis of DEGs in GBM

**Fig. 3:**
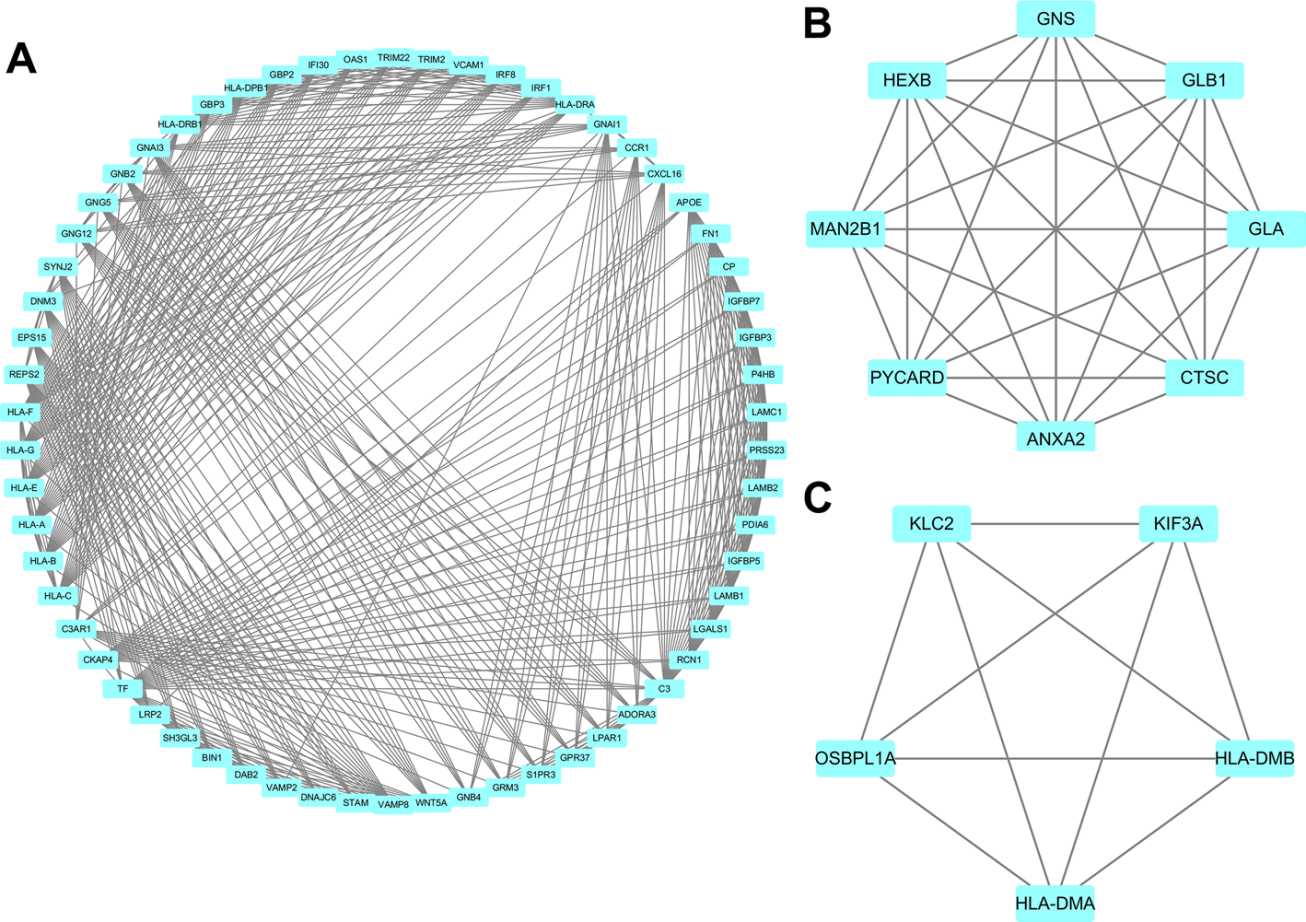
The protein–protein interaction (PPI) network and hub gene analysis of DEGs in GBM. **A** Module 1, **B** module 3 and **C** module 10 in the protein–protein interaction (PPI) network

All DEGs were subject to PPI network analysis utilizing STRING database [17] with the highest confidence (greater than 0.9). Next, the data file downloaded from STRING analysis was visualized utilizing Cytoscape software. Generally, the PPI network covered 324 nodes and 1284 edges. To identify the hub genes, the nodes with a connectivity degree greater than 10 in the whole PPI network were collected. A total of 94 genes were regarded as hub genes, including 73 upregulated genes and 21 downregulated genes. Furthermore, MCODE application results were indicative of three significant modules (node score cut-off = 0.2, k-core = 2, depth from seed = 100). All network scoring of the three modules were more than 5.0. Module 1, module 3 and module 10 were significant modules in the PPI network. A total of 62 nodes and 504 edges were included in Module 1 (Fig. 3A), and a total of eight nodes and 18 edges were included in Module 3 (Fig. 3B). In addition, there were five nodes and 10 edges in Module 10 (Fig. 3C).

### Identification of prognostic genes

**Fig. 4:**
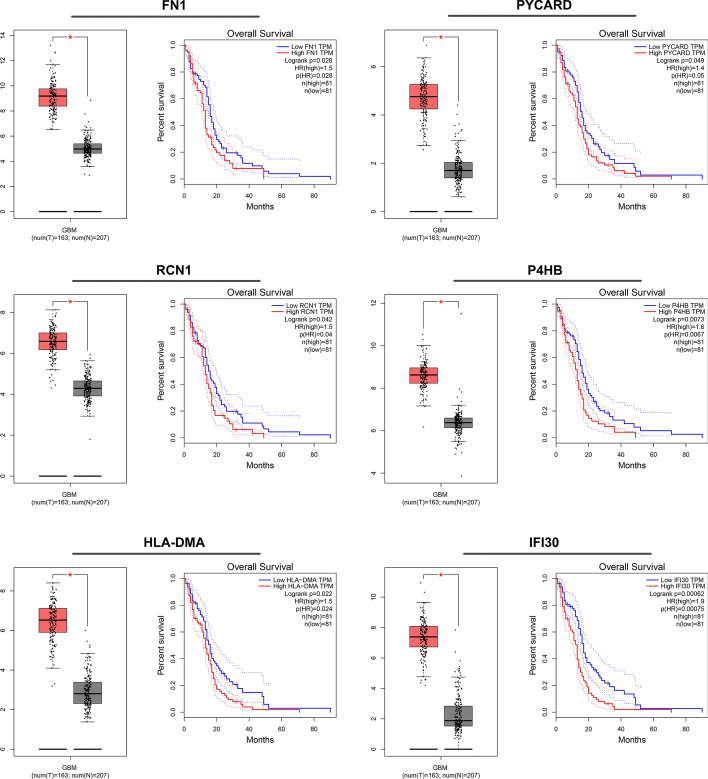
Confirmation of hub gene expression in GBM tissues. Gepia online tool was utilized to analyze the expression levels of six hub genes (FN1, PYCARD, RCN1, P4HB, HLA-DMA and IFI30) in TCGA-GBM tumors (*n* = 163) vs TCGA normal + GTEx normal tissues (*n* = 207). Data were analyzed with one-way ANOVA. Kaplan–Meier overall survival (OS) curves comparing high and low expression group of hub genes in GBM patients

**Fig. 5:**
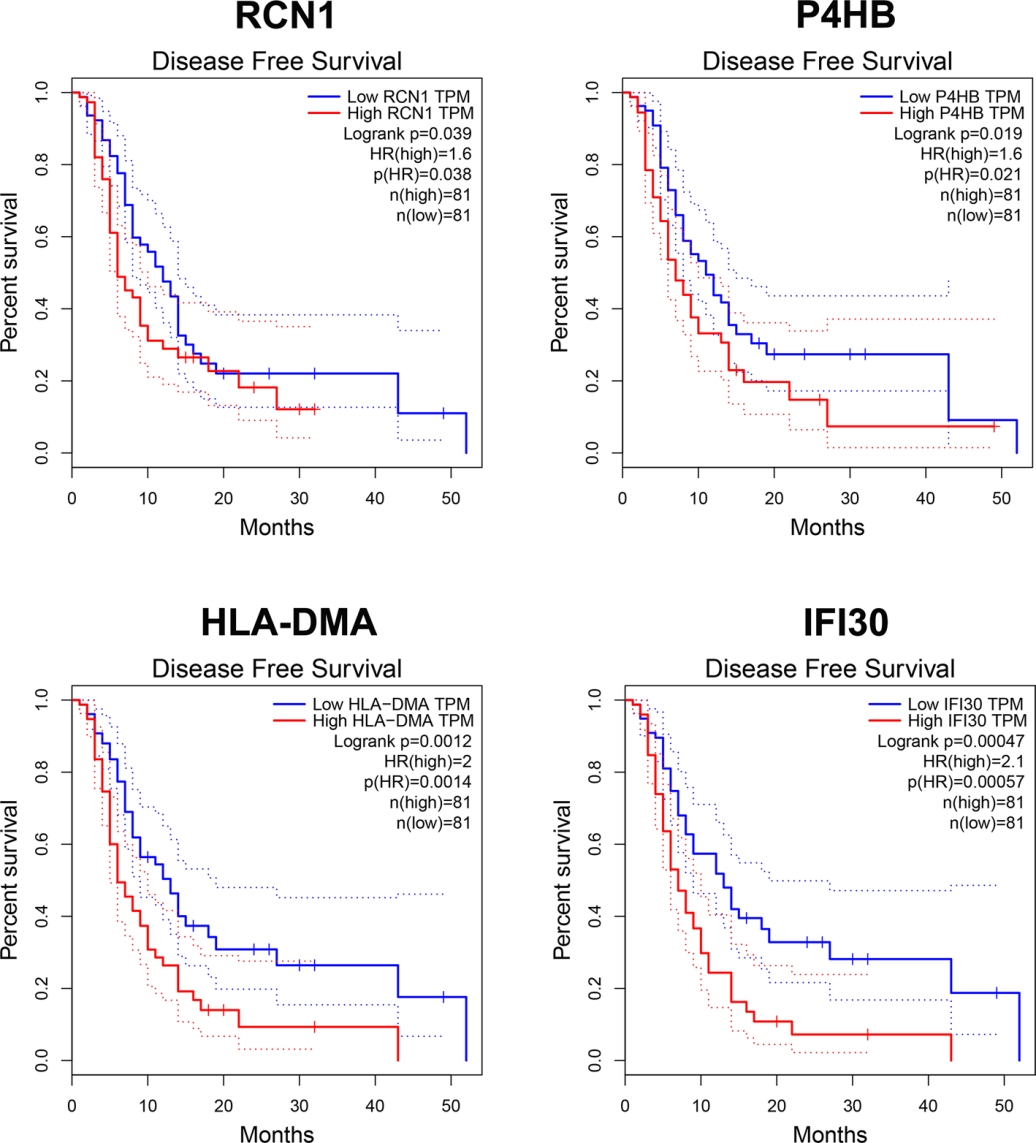
Identification of prognostic genes. Disease-free survival (DFS) of selected genes (RCN1, P4HB, HLA-DMA and IFI30) in GBM using TCGA database and Gepia online tool

To further identify the key prognostic genes, GEPIA online tool covering 163 GBM tissues and 207 non-tumor brain tissues data from the TCGA was utilized for the validation of hub gene expression. Moreover, the overall survival analysis was also performed. The results demonstrated that six hub genes not only were upregulated in GBM tissue samples (all *p* < 0.05), but also were markedly related to worse prognosis of GBM patients, including IFI30, HLA-DMA, P4HB, RCN1, FN1 and PYCARD (all *p* < 0.05, Fig. 4). In addition, among the six hub genes, the high levels of four genes were related to worse DFS of GBM patients (all *p* < 0.05, Fig. 5). These results indicated that IFI30, HLA-DMA, P4HB and RCN1 might be considered as oncogene and prognostic genes in GBM.

### Correlation analysis between prognostic genes

**Fig. 6:**
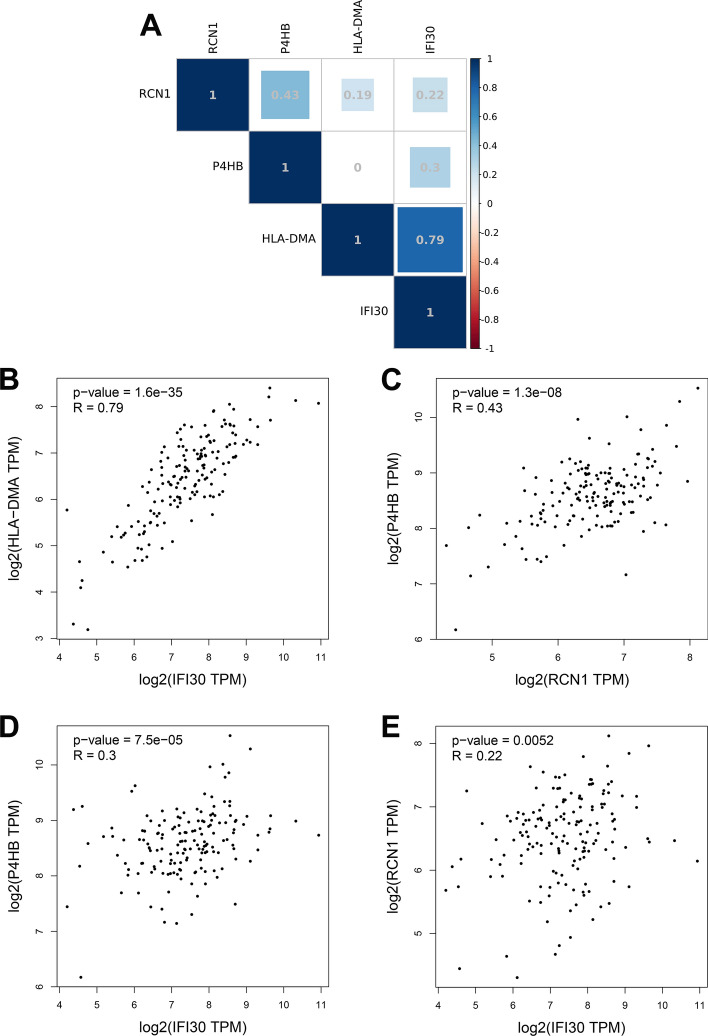
Correlation analysis between prognostic genes. **A** Heat map of the correlation between RCN1, P4HB, HLA-DMA and IFI30. The blue represents positive correlation. Spearman correlation analysis **B** between IFI30 and HLA-MDA (*R* = 0.79), **C** between RCN1 and P4HB (*R* = 0.43), **D** between IFI30 and P4HB (*R* = 0.3), *E* between IFI30 and RCN1 (*R* = 0.22)

Spearman’s correlation analysis was carried out to analyze the expression correlation between the four prognostic genes (IFI30, HLA-DMA, P4HB and RCN1) (Fig. 6A). As displayed in Fig. 6B–E, the level of IFI30 was positively correlated with HLA-DMA (*R* = 0.79, *p* < 0.001), P4HB (*R* = 0.3, *p* < 0.001) and RCN1 (*R* = 0.22, *p* < 0.01). Additionally, the expression of RCN1 was positively correlated with P4HB (*R* = 0.43, *p* < 0.001) and HLA-DMA (*R* = 0.19, *p* < 0.01).

### Principal component analysis (PCA) verifies the grouping ability of selected prognostic genes

**Fig. 7:**
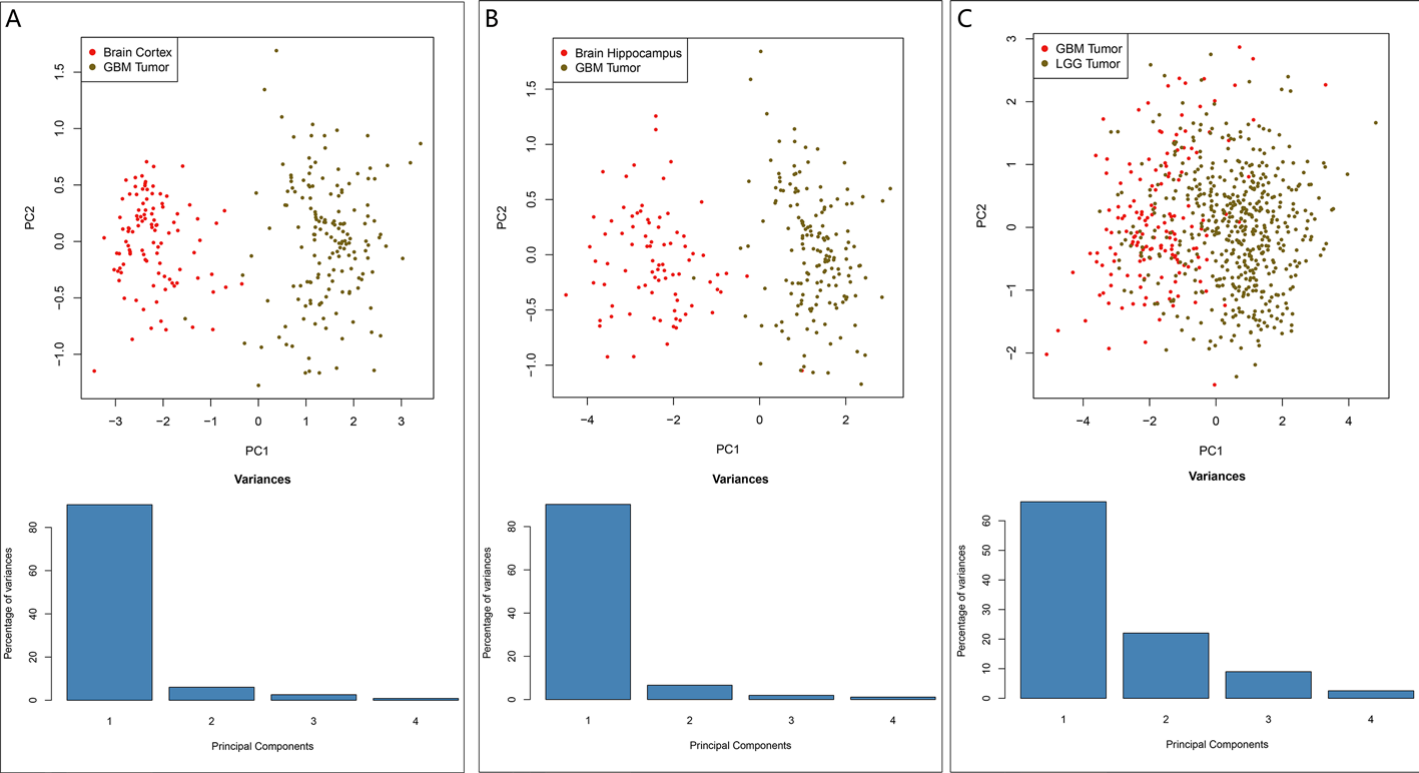
Principal component analysis between the GBM and LGG or non-tumor brain tissue groups based on screened prognostic-related genes. PCA 2D scatter plots and scree plots showed within-sample variation between GBM and brain-cortex (**A**), brain-hippocampus (**B**) or LGG (**C**) based on screened prognostic-related gene set. Each color of dot represents individual tumor

To reduce high-dimensionality genes with poor prognostic significance into a limited number of principal components, PCA was performed to detect the prognostic-related genes between the GBM and LGG or non-tumor brain tissues. PCA identifies new variables, the principal components, which are linear combinations of the original prognostic genes. The components have a sample-like pattern with a weight for each gene. The principal components are normalized eigenvectors of the covariance matrix of the genes and ordered according to how much of the variation present in the data they contain. Each component can then be interpreted as the direction, uncorrelated to previous components, which maximizes the variance of the samples when projected onto the component. Then we looked at the proportion of the variance present in all genes contained within each principal component. The result is that the dimensionality can be reduced from the number of genes down to two dimensions, while still retaining information that separates GBM from brain-cortex or LGG from GBM. The scatter plots indicated that principal components 1 (PC1) contributed to more than 80% of the variation in either group GBM vs brain-cortex or GBM vs Hippocampus. And the first two principal components were used to generate stable clusters. Figure 7 reveals that GBM group was well separated from either brain-cortex or hippocampus in the first principal component. However, PCA did not separate samples based on GBM and LGG clearly.

### Expression analysis of prognostic genes by qRT-PCR and immunohistochemistry

**Fig. 8:**
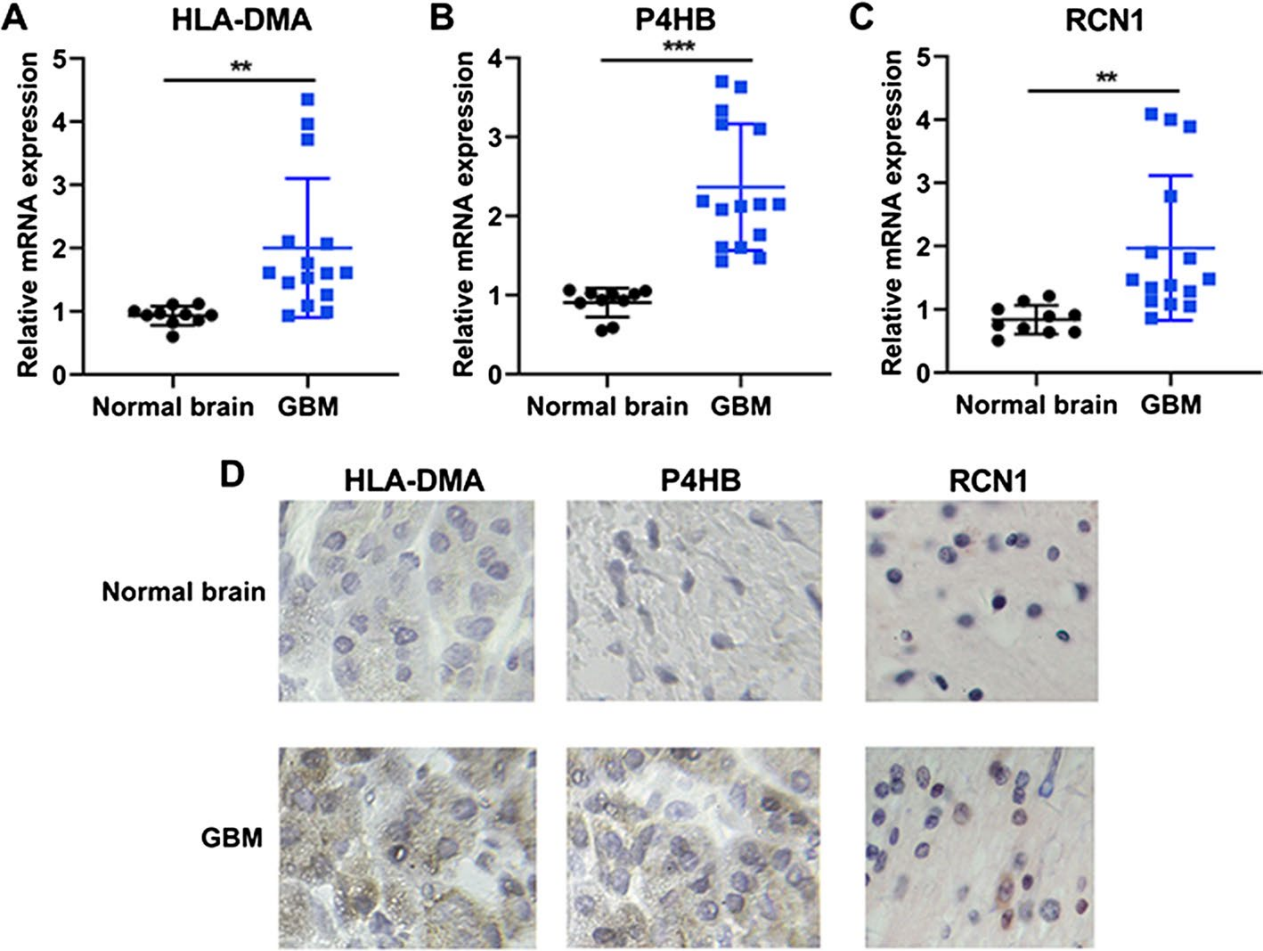
The expression of P4HB, HLA-MDA and RCN1 in GBM and normal brain tissues. **A**–**C** qRT-PCR analysis of P4HB, HLA-MDA and RCN1 mRNA expression in GBM tissues (*n* = 15) and normal brain tissues (*n* = 10). Data were showed as mean + SD and analyzed with unpaired *t* test. **p < 0.01, ***p < 0.001. **D** Immunohistochemical analysis of P4HB, HLA-MDA and RCN1 expression in GBM. **E** IHC score in GBM tissues and normal brain tissues. Data were showed as mean + SD and analyzed with two-way ANOVA, followed by Bonferroni's multiple comparisons test. ***p < 0.001

Clinical study has confirmed that IFI30 expression was upregulated in GBM tissues and was a significantly poor prognostic marker of patients with glioma [18, 19]. However, the expression HLA-DMA, P4HB and RCN1 in GBM was not elucidated. In this research, we further confirmed the levels of the above prognostic genes in GBM tissue samples and normal brain specimens by qRT-PCR analysis and IHC assay. Of the mRNA levels analyzed, HLA-DMA, P4HB and RCN1 demonstrated differential expression between GBM tissues and normal brain samples (all *p* < 0.001, Fig. 8A–C). Moreover, we performed IHC analysis to complement our qRT-PCR result as well as to detect protein expression in GBM tissues. The IHC analysis displayed that GBM tissues were expressing significant high levels of HLA-DMA, P4HB and RCN1 (Fig. 8D). Due to the specificities of antibodies, IHC analysis is required for identification and evaluation of cancer biomarkers, to explore protein function and verify the accuracy of prognostic tests.

## Discussion

Despite the effective conventional GBM therapeutics clinically, several patients with GBM suffer from cancer recrudesce and metastasis, as an aggressive malignant brain tumor in CNS [20]. Therefore, it is crucial to explore regulators driving GBM tumor development and to develop biomarkers that can better predict postoperative patients with aggressive GBM.

Bioinformatics methods have emerged since the advent of high-throughput sequencing and microassay technology. Moreover, several studies have utilized TCGA or GEO datasets to identify numerous biomarkers related to worse prognosis and survival in GBM [21, 22]. In the present research, we thoroughly identified and analyzed a total of 719 DEGs from two microarray datasets. Ninety-four hub genes were found in the PPI network while six genes were interestingly found in the independent expression test of the TCGA database. Additionally, it was confirmed in clinical tissues that the four genes (RCN1, P4HB, HLA-DMA and IFI30) were overexpressed in GBM tissues compared with normal brain tissues.

IFI30 is considered to be a typical regulating immune regulator belonging to the γ-interferon stimulated gene family [19], responsible for the activation of tumor progression. Its role in the development of glioma mainly depends on its coding protein γ-interferon-inducible lysosomal thiol reductase (GILT) [23]. Liu et al. detected the expression of IFI30 in glioma with wild-type isocitrate dehydrogenase (IDH) and the infiltration response in immune cells by TCGA datasets analysis [18]. They found that the level of IFI30 was higher in GBM specimens than in low-grade glioma (LGG) specimens. Moreover, its expression was also increased in wild-type IDH samples [18]. Interestingly, the analysis indicated that the upregulation of IFI30 contributed to worse prognosis and immunosuppression which triggered extracellular matrix dysfunction and angiogenesis in GBM [18]. However, the exact molecular interactions that associated with IFI30 in GBM procession remain less clear.

The results in this study verified that the expression of IFI30 was positively associated with the mRNA expression pattern of HLA-DMA, P4HB and RCN1. Previous studies suggested that the three genes (HLA-DMA, P4HB, RCN1) whose expression was related to IFI30 level in GBM exerted tumorigenic functions in malignant phenotypes. HLA-DMA is mainly associated with breast cancer [24]. It indicated that tumors that express HLA-DMA might contribute to the enhanced levels of immunity response and patient outcome [24]. P4HB is an autophagy-related gene which plays a role in diagnosis of kidney renal clear cell carcinoma [25]. In addition, liver cancer cells with overexpressed P4HB favored cancer cell growth and epithelial–mesenchymal transition, and also enhanced cancer cell chemoresistance [26]. RCN1 is upregulated in oral squamous cell carcinoma, prostatic cancer and non-small cell lung cancer [27–29]. RCN1 served as a biomarkers for tumor diagnosis and prognosis.

The most significant correction is IFI30 vs HLA-DMA. As a peptide processor, HLA-DMA catalyzes peptide conversion on classical MHCII proteins [30] and protects empty MHC class II molecules from functional inactivation during efficient presentation of protein antigens [31]. HLA-DMA has been known to be involved in cancer progression and drug-resistance [32]. There is an evidence that the enrichment of HLA-DMA in gastric cancer RNAseq expression profiling involves the activation of antigen processing and presentation pathway [33]. Another research reported that increased level of HLA-DMA is related to immune response in GBM, as a novel target for GBM diagnosis and treatment [34]. We gave a new confirmation for that HLA-DMA is expected to become a biomarker for GBM prognosis.

P4HB is an endoplasmic reticulum chaperone protein with overexpression in numerous tumor tissues [35]. P4HB has a significant prognostic potential in bladder urothelial carcinoma by serving as an autophagy-related gene [36]. Consistent with the prominent role of P4HB during tumorigenesis, upregulation of P4HB expression is frequently observed during cell resistance to drug. It has been shown that inhibition of P4HB expression in glioma cells results in suppression of temozolomide resistance by regulating endoplasmic reticulum stress response [37]. In combination with the view that immune response in cellular stress is involved in dysfunction of endoplasmic reticulum [38], this study gave a predicting result that IFI30, a potential immune-associated target in GBM, was positively correlated with P4HB level.

RCN1, a monomeric cell surface-associated protein encoding an endoplasmic reticulum (ER)-resident Ca^2+^-binding domain, plays a crucial role in cancer metastasis and development [39]. Although RCN1 is expressed in endothelial cells, the increase of RCN1 expression is indicative of tumor characteristics [40]. A recent study found that in nasopharyngeal carcinoma with sensitivity Adriamycin, the down-expression of RCN1 significantly enhanced cell drug-sensitivity [41]. In addition, RCN1 is molecular marker for the diagnosis and prognosis of non-small cell lung cancer [29]. Our data indicated that RCN1 has impact on OS and DFS rate of GBM patients for the first time and its potential oncogenic function would be related to IFI30 expression. It would be meaningful to confirm exact function of RCN1 in GBM in future.

Subsequently, we collected several clinical GBM tumor tissues and detected the expression levels of HLA-DMA, P4HB and RCN1 in GBM via qRT-PCR and IHC assays. The results indicated that HLA-DMA, P4HB and RCN1 expression was upregulated in GBM. However, clinical and therapeutic evidences that the genes function as prognostic are lacking. More follow-up studies will be performed to verify the prognostic role of these genes in patients with GBM. In fact, studies have reported that IFI30 and HLA-DMA could be related to immune response process [24, 42] which play tumor-regulatory roles in GBM [43]. The association between IFI30 or HLA-DMA and GBM immune microenvironment will be the topic of further research. In addition, recent reports have revealed that P4HB and RCN1 acted as tumor-suppressor factors in liver cancer [26], colon cancer [44], lung cancer [29] and prostate cancer [28]. In the future study, we may combine gene expression levels of P4HB or RCN1 and cell biological behavior analysis to further explore the pathogenesis of GBM.

## Conclusions

This study identified four significant DEGs (IFI30, HLA-DMA, P4HB and RCN1) between GBM and normal brain tissues via analyzing two microarray datasets. The identification of novel biomarkers may be helpful to improve the clinical outcome of GBM patients and provide a combined approach. These four genes are potentially regarded as novel biomarkers for prognosis in GBM. It will contribute to the identification of GBM patients and probably result in a convenience of monitoring outcome of patients with GBM. However, this study had some limitations due to the limited sample size obtained from microassay datasets and the lack of survival analysis on adequate clinical samples. Future prospective research is indispensable to include a larger sample size and evaluate the clinical values of the four GBM biomarkers.

## Methods

### Microarray data and DEGs identification

GSE15824 and GSE16011 profiles were downloaded from the GEO database (https://www.ncbi.nlm.nih.gov/geo/). The GSE15824 dataset was generated utilizing the GPL570 [HG-U133_Plus_2] Affymetrix Human Genome U133 Plus 2.0 Array platform and contained two non-tumor samples and twelve GBM samples (12 primary and 0 secondary). The GSE16011 dataset was generated using the GPL8542 Affymetrix GeneChip Human Genome U133 Plus 2.0 Array platform and contained eight non-tumor samples and 159 GBM samples (106 primary and 53 secondary). Limma package (version: 3.40.2) of R software and GEO2R online analysis was utilized to identify DEGs. The false positive results in GEO datasets were corrected by the adjusted p-value. Adjusted *p* < 0.05 and log_2_ (fold Change) (log_2_|FC|) > 2 were identified as the thresholds for the screening of DEGs.

### GO and KEGG pathway enrichment analysis

To comprehensively investigate the biological meaning behind DEGs intersection, the Database for Annotation, Visualization and Integrated Discovery (DAVID) (https://david.ncifcrf.gov/) [45, 46] was used to obtain the set of functional annotation. GO (http://www.geneontology.org/) is an extensively used tool for annotating genes with potential functions, such as molecular function (MF), biological pathways (BP) and cellular components (CC). KEGG enrichment analysis (https://www.kegg.jp/) is a practical resource for analytical study of gene functions and associated high-level genome functional information. *p* < 0.05 was regarded as the threshold value.

### PPI network and modules analysis

The Search Tool for the Retrieval of Interacting Genes (STRING, http://string-db.org) is a public resource designed to analyze PPI information. In the present research, the STRING online tool was utilized to generate the full STRING network containing all DEGs. The required score (highest confidence) was set as greater than 0.9 to validate significant interactions. In addition, the STRING network file was downloaded and subject to visualization using Cytoscape software (version 3.5.1) [47]. The nodes with a degree greater than 10 were regarded as hub genes and applied for further analysis. Furthermore, the clusters in 94 hub genes were constructed using Molecular Complex Detection (MCODE) application (version 2.2) with Node score cut-off = 0.2, k-core = 2 and max depth = 100.

### Expression levels and survival analysis of Hub genes in GBM

The gene expression levels and GBM patient survival were analyzed using the Gene expression profiling and interactive analyses (GEPIA, http://gepia.cancer-pku.cn/) [48]. The GEPIA contained 162 GBM samples and 207 non-tumor samples and the expression of hub genes was analyzed. In addition, the prognosis information of GBM patients, including OS and disease-free survival (DFS), was also collected to identify the prognostic biomarkers among hub genes.

### GBM tissues collection and immunohistochemistry (IHC)

This study was approved by the ethics committee of Second hospital of Shanxi Medical University. A total of 15 tumor samples were collected from patients with GBM after obtaining written informed consent between June 2016 and May 2020. All patients were diagnosed with GBM by two experienced pathologists and all patients did not undergo any treatment before surgery. A total of 10 normal brain tissues were isolated from patients with epilepsy. The sections of 4-μm thickness were collected from GBM and normal brain tissues, immobilized with 4% paraformaldehyde for 15 min, and then incubated with 0.3% H_2_O_2_ solutions for 20 min. After that, the sections were incubated with 5% BSA at room temperature to inhibit nonspecific binding. Anti-HLA-DMA (sc-134356, Santa Cruz Biotechnology, Santa Cruz, CA, USA), anti-P4HB (ab137110, Abcam, Cambridge, UK) and anti-RCN1 (ab210404, Abcam) antibodies were used to stain with the specific proteins. The slides were washed four times, incubated with biotinylated secondary antibody for 1 h, and incubated with streptavidin-HRP reagent for 10 min. Next, the sections were placed in DAB kit (ab64238, Abcam) for incubation of 10 min. The images of tissues were captured with a microscope.

### RT-qPCR

Total RNAs were isolated from GBM tissues using TRIzol reagent (Invitrogen, Carlsbad, CA, USA). First-Strand Synthesis System Kit (Invitrogen) was used for cDNA synthesis. Next, qPCR was performed using Applied Biosystems™ PowerUp™ SYBR™ Green Mix (Applied Biosystems, Carlsbad, CA, USA) and specific primers. Primer sequences for target genes and reference genes are listed as following. HLA-DMA, forward primer: 5ʹ-ATG GGA AAA TCC CGG TGT CC-3ʹ, reverse primer: 5ʹ-GTT GTG ATA GGC AGG CCA CT-3ʹ; P4HB, forward primer: 5ʹ-TGC TGC GGA AAA GCA ACT TC-3ʹ, reverse primer: 5ʹ-GTC ATC TCC TCC TCC AGG GT-3ʹ; RCN1, forward primer: 5ʹ-ACG AGA GCA AGG AGA GGC TA-3ʹ, reverse primer: 5ʹ-TCC TTC CAG ACT TTG GCG AC-3ʹ; GAPDH, forward primer: 5ʹ-GCT CTC TGC TCC TCC TGT TC-3ʹ, reverse primer: 5ʹ-TTC CCG TTC TCA GCC TTG AC-3ʹ. qPCR assays were performed on the iQ5 Real-time PCR system (Bio-Rad, Hercules, CA, USA).

### Correlation analysis

GEPIA online tool was utilized for analyzing the correlation between the hub genes with Spearman's correlation analysis. *p* < 0.05 was considered as significant difference.

## Data Availability

The open-access datasets are available through the following URL: GSE15824 (https://www.ncbi.nlm.nih.gov/geo/query/acc.cgi?acc=GSE15824) and GSE16011 (https://www.ncbi.nlm.nih.gov/geo/query/acc.cgi?acc=GSE16011). All data generated or analyzed during this study are available from the corresponding author on reasonable request.

## Abbreviations

GBM: Glioblastoma
DEGs: Differentially expressed genes
GO: Gene Ontology
KEGG: Kyoto Encyclopedia of Genes and Genomes
PPI: Protein–protein interaction
STRING: Search Tool for the Retrieval of Interacting Genes
GEPIA: Gene expression profiling and interactive analyses
IFI30: Interferon-gamma-inducible protein 30
HLA-DMA: Major histocompatibility complex class II-DM alpha
P4HB: Prolyl 4-hydroxylase beta polypeptide
RCN1: Reticulocalbin-1
CNS: Central nervous system
OS: Overall survival
EGFR: Epidermal growth factor receptor
IDH: Isocitrate dehydrogenase
MGMT: O6-Methylguanine-DNA methyltransferase
GEO: Gene Expression Omnibus
DAVID: The Database for Annotation, Visualization and Integrated Discovery
MF: Molecular function
BP: Biological pathways
CC: Cellular components
MCODE: Molecular Complex Detection
DFS: Disease-free survival
IHC: Immunohistochemistry
IDH: Isocitrate dehydrogenase
LGG: Low-grade glioma
